# Musculo-skeletal Ultrasound in paediatric rheumatology: experience of one University Hospital

**DOI:** 10.1186/1546-0096-9-S1-P42

**Published:** 2011-09-14

**Authors:** J Madruga Dias, M Manuela Costa, F Saraiva, JA Pereira da Silva

**Affiliations:** 1 Department of Rheumatology, Hospital de Santa Maria, Lisbon, Portugal

## Introduction

Musculo-skeletal Ultrasound (MSK-US) has become essential in the diagnosis and follow-up of children with rheumatic diseases. It’s an innocuous exam that helps diagnostic and treatment decisions and allows treatment efficacy assessment.

## Objectives

To evaluate the usefulness of MSK-US in the study of paediatric rheumatic inflammatory diseases.

## Material and methods

Analysis of 330 MSK-US exams performed to 222 children with rheumatic inflammatory diseases in our Department. The children’s ages were between 1 and 18 years (average= 11,7±4,7 years) and 67,6% were female. Synovial membrane proliferation, intra-articular effusion, cartilage contour abnormalities, erosions and periarticular affections were searched in each joint. Clinical and ultrasonographic data were correlated.

## Results

MSK-US detected synovitis in 100 of 194 exams (51,5%) of patients that had clinical suspicion of arthritis and in 36 of 136 exams (26,5%) of patients that presented with another symptom. In patients with clinical, but not ultrasonographic synovitis (94), we detected tenosynovitis in 13 cases (13,8%) and synovial cyst in 4 (4,3%). The remaining patients had no ultrasonographic changes.

Overall 39 exams showed ultrasonographic tenosynovitis, 15 (38,5%) of which had the clinical diagnosis too. MSK-US also identified erosions in 7 patients (2 had no ultrasonographic arthritis).

In total, 381 ultrasonographic diagnoses were made. Considering that 49,7% of the exams performed had no abnormalities, 1,3 diagnosis per exam were made.

## Conclusions

MSK US confirms or excludes arthritis, which permits a fast treatment or correct therapeutic options, avoiding iatrogenesis in children. It also allows the diagnosis of other articular and periarticular affections, and ultrasound-guided procedures.

